# Investigations on the occurrence of a muscular disorder in Austrian slaughter pigs

**DOI:** 10.1186/s40813-021-00230-1

**Published:** 2021-08-31

**Authors:** Lukas Schwarz, Carolin Schöner, Rene Brunthaler, Herbert Weissenböck, Tanja Bernreiter-Hofer, Barbara Wallner, Andrea Ladinig

**Affiliations:** 1 Department for Farm Animals and Veterinary Public Health, University Clinic for Swine, University of Veterinary Medicine, Vienna, Vienna, A-1210 Austria; 2 Department of Pathobiology, Institute of Pathology, University of Veterinary Medicine, Vienna, Vienna, A-1210 Austria; 3 Department of Biomedical Science, Institute of Animal Breeding and Genetics, University of Veterinary Medicine, Vienna, Vienna, A-1210 Austria

**Keywords:** Dystrophin, Fatty muscular dystrophy, Muscular steatosis, Pig

## Abstract

**Background:**

In slaughterhouse, veterinarians responsible for meat inspection are often confronted with abnormalities in carcasses, not only in pigs but in all livestock species. In 2017, a veterinarian responsible for meat inspection in a slaughterhouse in Styria, Austria, observed gluteal muscles infiltrated by fat and muscle tissue obviously being replaced by fat in two different slaughter pigs. These muscles were sent for further diagnostics to the University Clinic for Swine.

**Results:**

The two muscle samples were investigated histopathologically and diagnosed with fatty muscular dystrophy. The results of routine histopathology were confirmed by dystrophin-specific immunohistochemistry. Sex of the two affected animals was determined retrospectively using a PCR-based protocol and resulted in one male and one female pig. A survey to determine the prevalence of fatty muscular disorders of pork revealed that this phenomenon gets frequently observed in Styria, but also occurs in Upper Austria and Lower Austria. Mostly gluteal and lumbal muscles were affected and approximately 20–40% of the affected muscles were replaced by fat.

**Conclusions:**

Fatty muscular dystrophy or muscular steatosis, as it was sometimes called in early literature, seems not to be an uncommon and rare event and is known to have several different causes. As it was detected in both sexes, our observations are different to the described case in Japan, where only one male individual was affected. To avoid further increase of such cases (fatty muscular dystrophy), it would be useful to clarify the cause. First, whether the cause is environmental or genetic, and in case it is genetic it would be key to disentangle the underlying genomic architecture. Having causal variants described—one could think about integrating this information (depending on the mode of inheritance and the number of loci involved) in the breeding program of pigs. Furthermore, the proportion of non-Austrian pig genetics used for commercial pig production in Austria should be reviewed in order to be able to make reliable statements about the spread of the disease not only in Austrian pig breeds, but also in pig breeds worldwide.

**Supplementary Information:**

The online version contains supplementary material available at 10.1186/s40813-021-00230-1.

## Background

Meat and carcass inspection at slaughterhouse is sometimes challenging for responsible veterinarians, as abnormalities and lesions of organs and tissues cannot always be assessed macroscopically. Once a rare and undetermined abnormality occurs, meat inspecting veterinarians should put effort in finding the cause of this abnormality. Especially muscular abnormalities may stress veterinarians in making decisions whether to discard the carcass or affected muscle parts or not. Using simple methods, such as histology followed by literature search may give valuable hints for uncommon and rare muscular abnormalities. However, the cause of the lesions must be investigated using molecular and genetic approaches.

In humans, but also in dogs, rodents and cats, dystrophic muscular diseases are well described and investigated [1–4]. The two forms, Duchenne Muscular Dystrophy (DMD) and Becker Muscular Dystrophy (BMD), are distinguished by the severity of the clinical course of affected patients [1]. While clinical symptoms of DMD, such as starting signs of locomotive disorders and immobility, start very early in life, symptoms of BMD usually emerge later and have a milder course, which leads to a longer survival of the affected individuals [1]. For diagnosing both forms of the disease, a combination of disease-related pedigree records and laboratory tests is available. Additionally, a muscle biopsy may be investigated for the presence and abundance of dystrophin, either immunohistochemically or via Western-blot [3, 4]. For studying DMD and BMD of humans, animal models were established in mice and Golden Retriever dogs [4].

In veterinary medicine, dystrophic muscular diseases affect different species. Although the mouse model is the most common animal model used for human research, pigs are more comparable to the human body [5]. Therefore, a BMD pig model was developed and can be used for studying disease progression and future therapy development [6]. Another research group established an alternative pig DMD model by genetically modifying the exon 52 of the dystrophin gene [7].

Despite dystrophic muscular diseases exist in domestic pigs, it is not common to spontaneously observe this pathological phenotype in pigs intended for meat production. This is because commercially used pigs do not reach the age at which pathological and clinical abnormalities are usually observed. Historically, there are several pathological descriptions of abnormalities of striated muscles in pigs, which probably all relate to the same or a similar clinicopathological entity: muscular steatosis, progressive primary myopathy, pseudohypertrophic atrophy, lipomatous pseudohypertrophy, interstitial lipomatosis, lipomatous muscular dystrophy, myosclerosis, hyperplasia or atrophia lipomatosa [8]. Underlying causative mechanisms are poorly understood and literature is very rare. However muscular steatosis may occur not only as the consequence of genetic disorder but as the result of chronically damaged or denervated muscles [9].

In 2013, Japanese researchers published a case of BMD-like myopathy in a slaughter pig. Further investigations using immunohistochemical staining of dystrophin, PCR-based sex determination and histopathological examination confirmed this case to be homologous to human BMD as the affected pig was a male [10]. Sex determination was performed, as the authors suggested a hereditary disease in pigs similar to human BMD, which is linked to the human X-chromosome [10, 11].

In 2019, a veterinarian responsible for meat inspection at a Styrian slaughterhouse observed severe abnormalities of gluteal muscles in two slaughter pigs from a single farm. These muscles were sent to the University Clinic for Swine, University of Veterinary Medicine Vienna, Vienna, for further investigations. The aim of this study was to gain information on muscular abnormalities in slaughter pigs in Austria based on histopathological and genetic investigations of muscular tissue from the two affected pigs from Styria as well as a survey across several meat inspecting veterinarians.

## Results

### Histological, immunohistochemical investigations and PCR-based sex determination

**Fig. 1:**
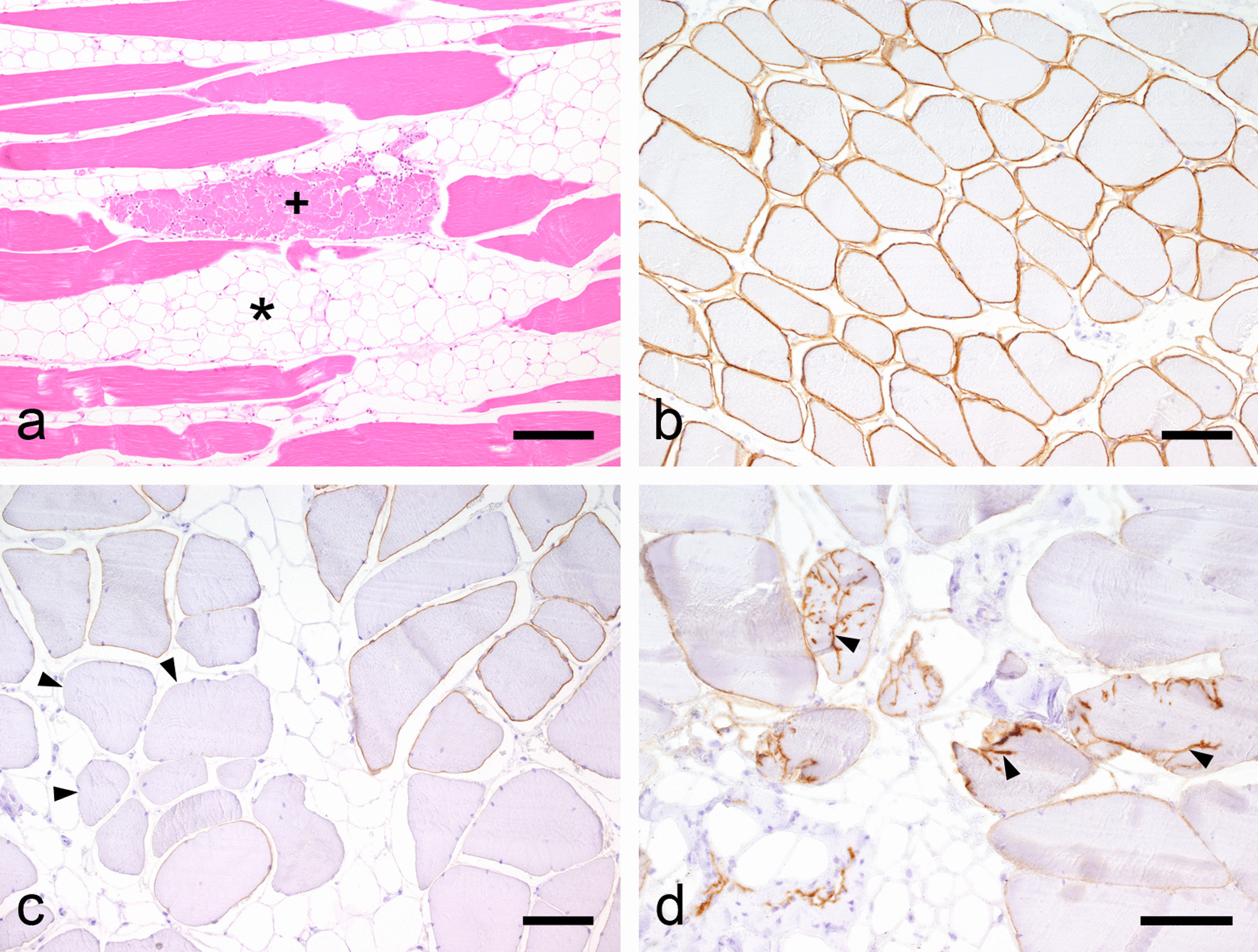
Histopathological and immunohistochemical investigations. **a** Skeletal muscle of a pig with fatty muscular dystrophy with diffuse infiltration of the interstitium (asterisk) with fat tissue and single fragmented muscle fiber (cross) (Hematoxylin and eosin (H&E), bar = 160 µm). **b** Immunhistochemical staining of unchanged skeletal muscle of a control pig with continuous dystrophin expression in the sarcolemma (Immunohistochemistry (IHC), bar = 80 µm). **c** Immunhistochemical evidence of reduction to complete loss (arrowhead) of dystrophin expression in an affected pig (IHC, bar = 80 µm). **d** Dislocation of the dystrophin expression to the cytoplasm of muscle fibres (arrowhead) (IHC, bar = 80 µm)

Histologically, tissue samples from both affected pigs showed severe diffuse infiltration of the interstitium with adipose tissue. The diameters of muscle fibres were highly variable and groups of severely atrophic fibres were observed, especially at the borders to fat tissue. Very few fibres lost their striation and became homogenized and segmentally fragmented. In some muscle cells the nuclei became centralized. Sporadically mild non-purulent infiltration around small interstitial arteries was found (Fig. 1a). Compared to control sections (Fig. 1b), immunohistochemical staining of dystrophin revealed marked alterations of its expression in the sarcolemma (Fig. 1c), ranging from reduction to complete absence of immunostaining. In one of the muscles, dystrophin expression was focally dislocated to the cytoplasm of muscle fibres (Fig. 1d). Summarized, the results of histopathological and immunohistochemical examination strongly supported the diagnosis fatty muscular dystrophy.

According to results of the PCR-based sex determination one affected pig was male, the other one female.

### Survey of Austrian meat inspecting veterinarians

Fourteen veterinarians gave feedback on their observations about fatty muscular disorders using our survey. The survey resulted in a response rate of 14.9% (14/94). Results of the survey are shown in Table 1.

**Table 1:** Summary of the data collected via surveying

Region	VET	SH	Lesions	FO	Affected muscles	% fat infiltration	Clinical signs in live animals
ST	1	1	Yes	5x/year	G, L	20–40%	none
ST	2	1	Yes	4x/year	G, L	20–40%	none
ST	3	1	Yes	10x/year	G, L	40–60%	none
ST	4	1	Yes	1x/month	G, L	20–40%	none
ST	5	1	Yes	1-2x/month	G, L	20–80%	none
ST	6	2	No	–	–	–	–
ST	7	3	Yes	1x/year	G, L	20–40%	none
ST	8	3	Yes	1 × in 5 years	G, L	20–40%	Not evaluated
ST	9	4	Yes	Sporadically	D	Not evaluated	–
LA	10	5	Yes	2-3x/year	G	40–60%	Not evaluated
UA	11	6	Yes	Once	G	20–40%	Not evaluated
UA	12	7	No	–	–	–	–
UA	13	7	No	–	–	–	–
CA	14	8	No	–	–	–	–

ST, Styria; LA, Lower Austria; UA, Upper Austria; CA, Carinthia; VET, meat inspecting veterinarian; SH, slaughterhouse; Lesions, lesions of fatty muscular dystrophy observed; FO, frequency of observations; G, gluteal muscle parts; L, lumbal muscle parts; D, diaphragmatic muscles

Most frequently, fatty muscular disorders were reported in Styria (8 different vets in 4 slaughterhouse) based on the survey, but also veterinarians of Lower Austria and Upper Austria reported at least once an indication of fatty muscular dystrophy. Most often, fatty muscular dystrophy was reported in slaughterhouse 1, with frequencies of 4-times a year to twice a month. In the remaining slaughterhouses, observations of fatty muscular dystrophy lesions were observed sporadically or were even single observations. In most cases the relative extent of fat infiltration in affected muscles parts was 20–40%, in some cases up to 80%. Clinical abnormalities were not reported in any individual during inspection of live animals before slaughtering. Out of all responses, four veterinarians never had observed fatty muscular disorders. Unfortunately, we cannot determine whether all of the non-responding persons (80/94) never observed fatty muscular abnormalities or just were uninterested in participating in the survey. Over the past few years (2018–2020), approximately five million pigs were slaughtered per year in Austria [12].

## Discussion

The results of this study show for the first time the possible incidence of muscular dystrophic diseases in swine in Austria, based on observations by meat inspecting veterinarians in slaughterhouse, assuming all observations have the same aetiology. Comparing our findings with the results in a Japanese study [9], histopathological alterations of the two investigated muscles from Styria were similar to the ones described there [10]. In both of our cases, severe fatty infiltration of affected muscles, degenerative hyalination and centrally located cell nuclei could be observed. Furthermore, the reduced expression of dystrophin in the sarcolemma could be confirmed by immunohistochemistry, which let us hypothesize that observed abnormalities may have been caused by a dystrophinopathy. DMD could be excluded in the Japanese as well as in our study, due to the fact that dystrophin was not totally lacking and no malfunctions of the muscular apparatus of live animals could be observed [1, 10]. Our findings in the two pigs from Styria differ at least in parts from the Japanese findings as we could not detect myophagy and fibrosis and there were only scarce necrotic myofibres. This may be due to a different underlying mechanism causing muscular dystrophy in the Austrian compared to the Japanese case—either genetically determined or based on another yet unknown and maybe also non-genetic aetiology [9]. It has been shown, that even different mutations in the dystrophin gene can result in variable properties of the protein and accordingly diverse histologic characteristics. For example, in a minipig the mutation of the central rod domain of the dystrophin gene led to mild clinical signs [6], whereas a modification of the gene at exon 52 in another pig resulted in severe DMD [7]. Moreover, a case in the United States reported a mutation of exon 41 of the dystrophin gene in a pig which, additionally to classical findings of muscle dystrophy of striated muscles, showed porcine stress syndrome [13]. As we do not know the cause of fatty muscular disorders in Austrian pigs, we can just speculate why our cases differ from the Japanese findings. Summarizing all recently described cases, obviously more than one mechanism exists which may end up in different muscular dystrophy or fatty muscular dystrophy, respectively.

Fatty muscular dystrophy is often caused by mutations in the dystrophin gene, which in humans as in pigs is located on the X-chromosome. X-chromosomal location of a gene causes a gonosomal mode of inheritance [10]. Assuming also a disease causing alteration in the dystrophin gene, we speculate that sows which carried an impaired X-chromosome could have resulted in affected piglets even if they were inseminated with semen of unaffected (homozygous for the wildtype allele) boars due to the gonosomal recessive inheritance which consequently developed fatty muscular dystrophy. Such matings would result in a majority of healthy siblings, but theoretically 25% of the male piglets may also end up in fatty muscular dystrophy, despite the boar was no carrier of a dystrophin gene mutation. Horiuchi et al. [10] suggested, analogous to human BMD and based on just one investigated case, that only male pigs were affected. As in our case one male and one female pig were affected by fatty muscular dystrophy, we hypothesize, that the mother sow and boar used for insemination may have been carriers of an impaired X-chromosome. As we did not investigate the genetic background of the two Austrian pigs, we can just speculate whether the cause of this fatty muscular disorder was based on a hereditary disease or if it has an absolutely different aetiology such as chronic muscle damage or denervation [9]. However, the occurrence of possibly fatty muscular dystrophy in a female pig could be more important than expected. Supposing fatty muscular dystrophy in Austrian pigs is a hereditary disease based on a recessive mutation on the dystrophin gene in both, male and female pigs, this would be alarming as it would imply a high allele frequency in the Austrian swine population. Next it would be indicated to find out the origin of affected animals for the determination of the genetic background and the influencing environment. This may be followed by a deep analysis of inheritance and estimation of heritability of the trait. Based on the afore mentioned steps one may put effort in determining responsible loci in the genome for establishing a molecular genetic test which probably could be used in swine breeding for eradication of fatty muscular dystrophy.

It has to be stated, that the reported observations in the survey may have had different aetiological backgrounds, other than fatty muscular dystrophy, such as replacement of muscle tissue after trauma, pressure or denervation [9]. Therefore it just can be hypothesized, whether all observations reported by meat inspecting veterinarians were caused by a dystrophinopathy or not. However, the two individual muscles described here most probably were affected by fatty muscular dystrophy, which could be supported by the results of immunohistochemical staining of dystrophin. Based on the results of the survey it can be concluded, that a muscular disorder, similar to the one of the two described muscles in this case report, is not only a regional problem in Styria, as corresponding lesions were reported by meat inspecting veterinarians also in Lower Austria and Upper Austria. Nevertheless, most observations were reported from Styrian veterinarians. We assessed the reports from Styria as relevant, as all responding meat inspecting veterinarians regularly and more than once a week inspect meat. Interestingly, in slaughterhouse one, observations of animals affected by fatty muscular dystrophy were reported once to twice a month. Hypothesizing that Austrian observations of fatty muscular disorders result from a hereditary disease background, one may speculate that the higher incidence in Styria is due to the establishment of carriers in local gilt and boar breeding stock. For this reason, further research is necessary to investigate whether fatty muscular disorders in Austria are genetically determined and if so, the underlying genetic basis should be resolved to detect carriers in Austrian swine breeds which serve as multiplying factors. Moreover, first a cost–benefit-analysis has to be performed to find out whether fatty muscular disorders in Austria are an economical or health related problem of pigs that would justify the time and effort of further research.

Another explanation for the accumulated occurrence in Styria could be, that the relatively low response rate of our survey may have substantially biased the situation. Despite it seems that reported cases primarily originated from Styria, we can just hypothesize about the reason for this observation.

## Conclusion

A muscular disorder, very similar to a described case of fatty muscular dystrophy in a Japanese pig was diagnosed for the first time in Austria and fatty muscular disorders were more often observed in Styria compared to other federal states. As we just can speculate about the causative mechanism of this fatty muscular disorder and observed muscular disorders, an in-depth analysis is indicated to elucidate the prevalence of fatty muscular dystrophy not only in the Austrian swine population, but also in the pig population worldwide. Further studies are needed to investigate the cause for the observed lesions in order to develop strategies to eradicate fatty muscular dystrophy and/or other muscular disorders.

## Methods

This study was performed in two different parts. Part one contained in-depth diagnostics of skeletal muscle samples of two slaughter pigs from Styria and part two was a survey among meat inspecting veterinarians at Austrian slaughterhouses.

### Muscle samples from slaughter pigs

Two gluteal muscle samples were sent for further diagnostics to the University Clinic for Swine, University of Veterinary Medicine Vienna, Austria, by a veterinarian responsible for meat inspection. Cubic pieces with an edge length of approximately 1.5 cm of each muscle were placed in 10% buffered formalin for histological and immunohistochemical investigations. Additionally, tissue of each muscle was stored at -20 °C for potential further investigations.

### Histological and immunohistochemical investigations

After 24 h of fixation, the samples were embedded in paraffin wax, sectioned at ~ 3 µm and stained with hematoxylin and eosin. Immunostaining for dystrophin was performed in order to check for a dystrophinopathy as underlying cause of this condition. Muscular dystrophies are characterized by defects in the gene coding for dystrophin, a sarcolemmal-associated cytoskeletal protein. Affected cases usually show complete (DMD) or partial (BMD) absence of immunostaining [9]. Dystrophin immunohistochemistry with a monoclonal antibody (dilution 1:50; Lab Vision, Fremont, CA, USA) was performed with an automated immunostainer (Thermo Autostainer 360-2D; Thermo-Fisher Scientific, Fremont, CA, USA) using the Ultravision LP detection system (Thermo-Fisher) and DAB as chromogen (Thermo-Fisher). As positive control gluteal muscle from an unaffected pig was used. For negative control the primary antibody was replaced by the respective immunoglobulin isotype.

### DNA isolation and PCR-based sex determination

Genomic DNA was isolated from 25 mg muscle tissue using Nexttec DNA Isolationskit (Nexttec, Germany). PCR sex determination was performed as described by Blanes et al. [14] including positive controls from pigs with confirmed sex and a no-template control. PCR fragment sizes were visualized on a 1.5% Agarose gel containing Atlas clearsight DNA dye (Atlas, Estonia).

### Survey among Austrian meat inspecting veterinarians

For the determination of the frequency of fatty muscular dystrophy or muscular steatosis a questionnaire was prepared. This questionnaire was sent to meat inspecting veterinarians in Lower and Upper Austria, Carinthia and Styria. Additionally, the survey was extended to operators of slaughter houses and meat processing companies with the goal to forward the questionnaire to the responsible veterinarians. The survey was conducted from May 2019 until November 2019 and the questionnaire was sent in total to 94 different recipients via e-mail. To increase the return rate of filled questionnaires we decided to keep it short in regards to the numbers of questions and points asked. Due to data protection reasons no person-related data were collected. In case of submission of additional personalized data, this was anonymized. The questionnaire first asked if fatty muscular dystrophy has been observed at all. In case of affirmation, consecutive questions were on the frequency of the observations, which muscle parts have been observed to be affected, the relative extent of affected muscle parts and if any clinical signs were observed during clinical examination of live animals at the slaughterhouse (Additional file 1). For explanatory reasons a description of fatty muscular dystrophy or muscular steatosis respectively was sent with the questionnaire (Additional file 2). This was necessary to exclude the misinterpretation of other muscular diseases as fatty muscular dystrophy.

Data were only descriptively analysed using Microsoft Excel (Microsoft Office 365, USA) due to the low response rate.

## Supplementary Information

**Additional file 1.** Blank form used for the survey.

**Additional file 2.** Description of an correponding example of the described muscular disorder and information for participating meat inspecting veterinarians in the survey.

## Data Availability

All data generated or analysed during this study are included in this published article or are available as supplementary data.

## Abbreviations

BMD: Becker muscular dystrophy
DMD: Duchenne muscular dystrophy
H&E: Hematoxylin and eosin
IHC: Immunohistochemistry
PCR: Polymerase chain reaction
DNA: Deoxyribonucleic acid
