# Supplementary material for: Investigations on the occurrence of a muscular disorder in Austrian slaughter pigs
Source: Porcine Health Manag. 2021 Aug 31;7:51. doi: 10.1186/s40813-021-00230-1 (PMC8406747; doi:10.1186/s40813-021-00230-1)
Supplement: Supplementary file 1 — Additional file 1. Blank form used for the survey. [file 40813_2021_230_MOESM1_ESM.docx]

**Survey "Fatty muscular dystrophy in Austrian slaughter pigs"**

| Date |  |
| --- | --- |
| Name (voluntary declaration) |  |
| Slaughter house (voluntary declaration) |  |
| Federal state |  |
| Have you ever observed the described lesions? | o yes o no |

If yes:

| Estimated frequency of observation/s | Approximately _______times per month  Approximately _______times a year  Approximately _______times in the last 5 years |
| --- | --- |
| Affected muscles | o gluteal muscles  o lumbal muscles  o other muscles:________________ |
| Grade of fat infiltration in affected muscles in % | o < 20%  o 20%- 40%  o 40%- 60%  o 60%- 80%  o > 80% |
| Clinical observations during life inspection of slaughter pigs before slaughtering | o none  o lameness  o other: ______________________  o not assessed |
