# Supplementary material for: Investigations on the occurrence of a muscular disorder in Austrian slaughter pigs
Source: Porcine Health Manag. 2021 Aug 31;7:51. doi: 10.1186/s40813-021-00230-1 (PMC8406747; doi:10.1186/s40813-021-00230-1)

**Fatty muscular dystrophy**

Fatty muscular dystrophy is a progressive degenerative disease of striated muscles. Most probably due to a genetic disorder a dysfunction or a deviation of the size of the dystrophin occurs. This disease progresses analogous to the human Becker-Muscle-Dystrophy and seems to be X-chromosomal inherited.

As a consequence of the disease it comes to a progressive degeneration of the affected muscles and to a loss of muscle fibres. Lost muscle tissue is replaced by fat in a way that no atrophy can be observed visually. Due to infiltration and replacement of muscles with/by fat, muscles appear pale and depending on the severity more or less light, as represented by the following picture.

Muscle infiltrated by fat and muscle tissue replace by fat

Physiological muscle tissue

Severe infiltration of muscle tissue with fat


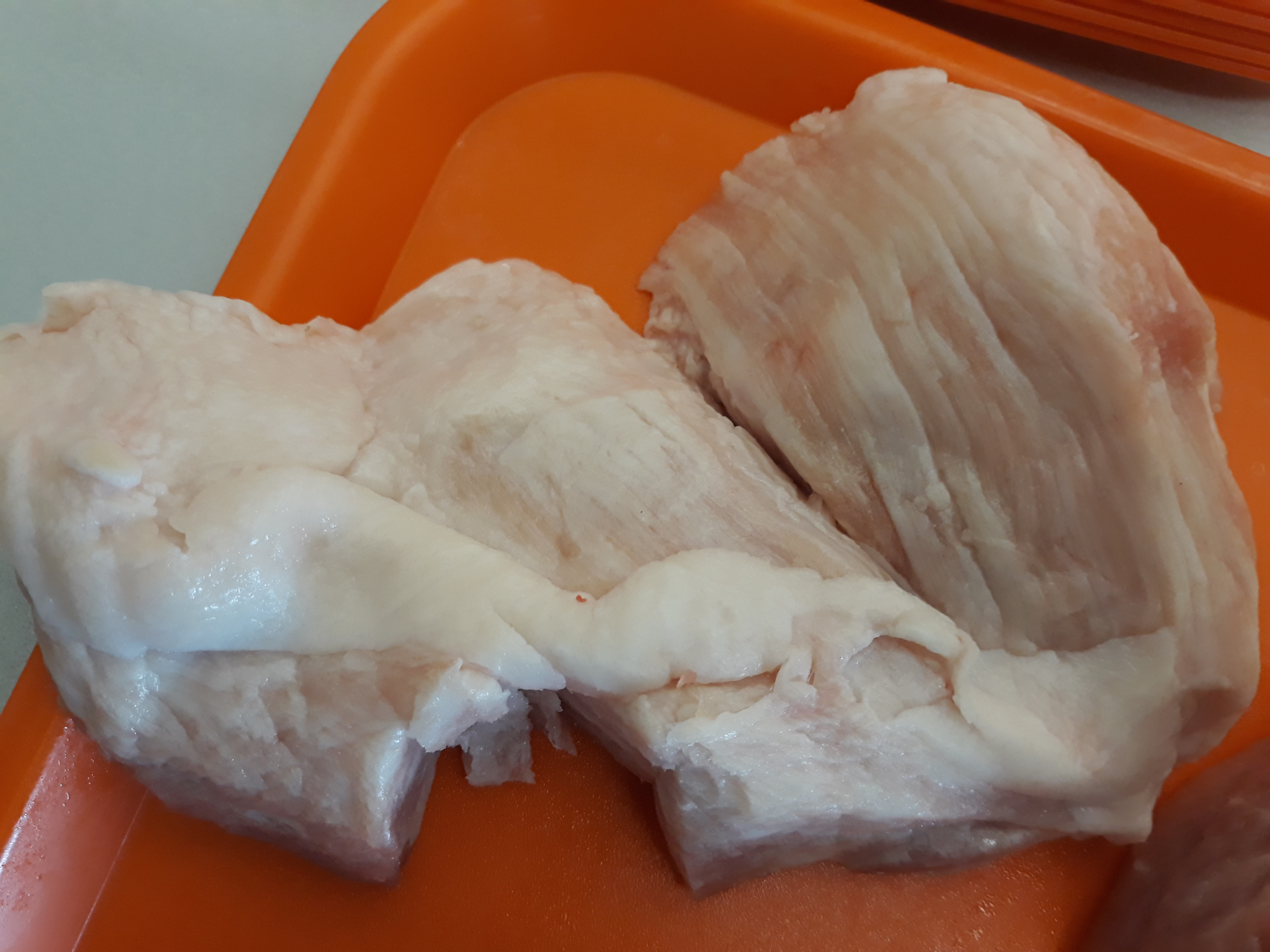


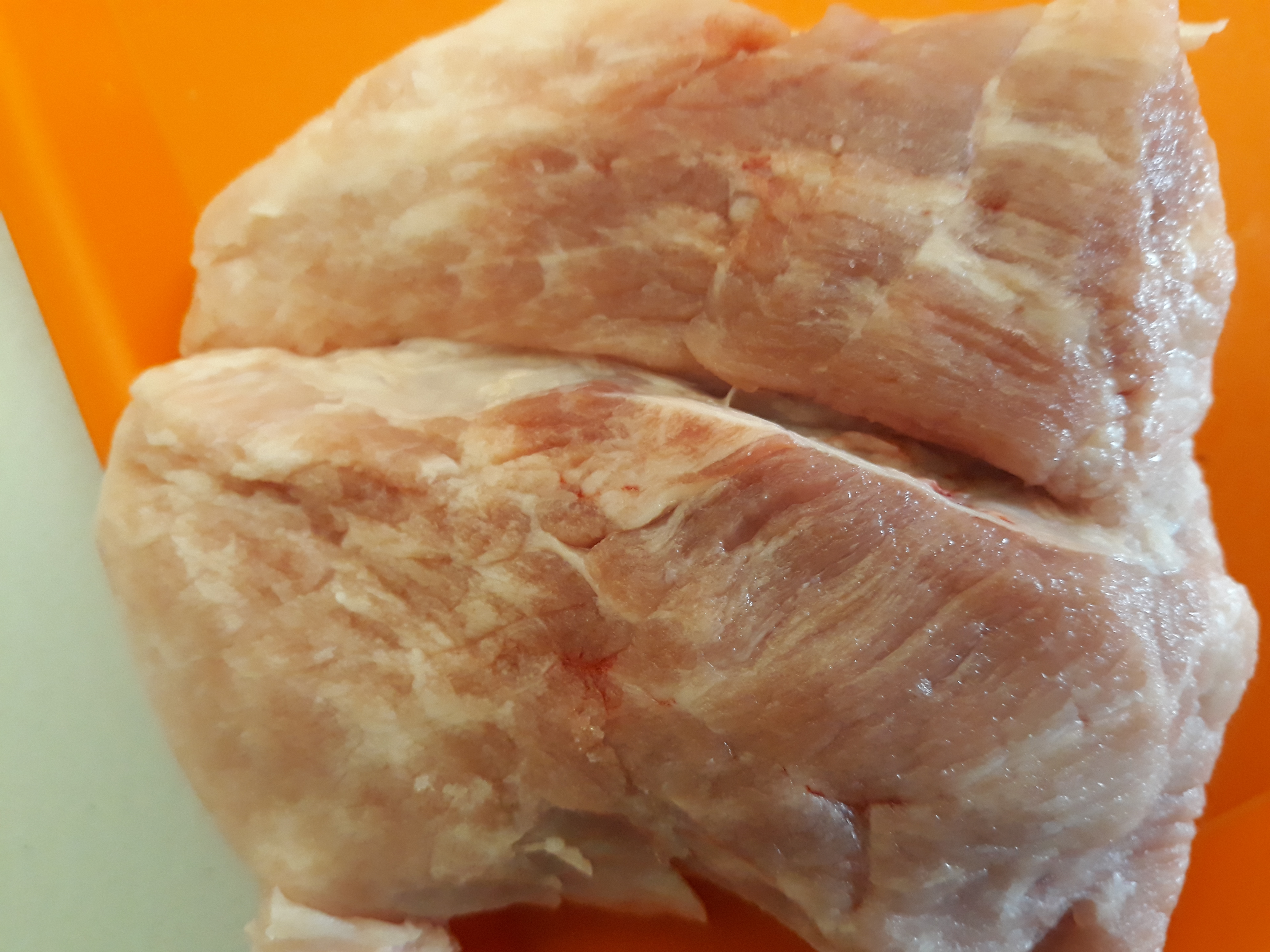

Supplement: Supplementary file 2 — Additional file 2. Description of an correponding example of the described muscular disorder and information for participating meat inspecting veterinarians in the survey. [file 40813_2021_230_MOESM2_ESM.docx]
